# Mesenchymal Hamartoma of the Liver: Complete Excision Always Necessary

**DOI:** 10.1155/2017/8314102

**Published:** 2017-11-16

**Authors:** Suman B. Koganti, Venu Madhav Thumma, Bheerappa Nagari

**Affiliations:** ^1^Department of Surgery, BLHC, Icahn School of Medicine at Mount Sinai, New York, NY 10457, USA; ^2^Department of Gastrointestinal Surgery, Nizam's Institute of Medical Sciences, Hyderabad 500082, India

## Abstract

Mesenchymal hamartoma (MH) is not an uncommon tumor of the liver in the age group of 2–10 years. It is the second most common benign liver tumor in children. Previously considered a developmental anomaly, newer insights into other theories of origin including toxic-metabolic, ischemic, and a true neoplastic process are in progress. Previous understanding of a purely benign nature of the tumor is being overridden by a real malignant transformation. Complete excision of the tumor with clear margins is recommended to achieve a long term cure. A thorough understanding of the natural history of these tumors and skillful surgical treatment are indispensable elements of care.

## 1. Introduction

Mesenchymal tumors of the liver are a common occurrence in the pediatric age group and are third in order following Hepatoblastoma and HCC. Mesenchymal hamartoma (MH) is the second most common benign liver tumor (next to Hemangioma) in the age group of 5–10 yrs and constitutes 30% of benign liver tumors and 5–8% of all the liver tumors in children [1–3]. Fewer than 200 cases are reported in the literature [1]. Several options for the management of these tumors including watchful waiting, enucleation, marsupialization, and liver resection are described [1]. We propose that complete excision of the tumor is safe and provides the best long term results.

## 2. Case Presentation

**Figure 1:**
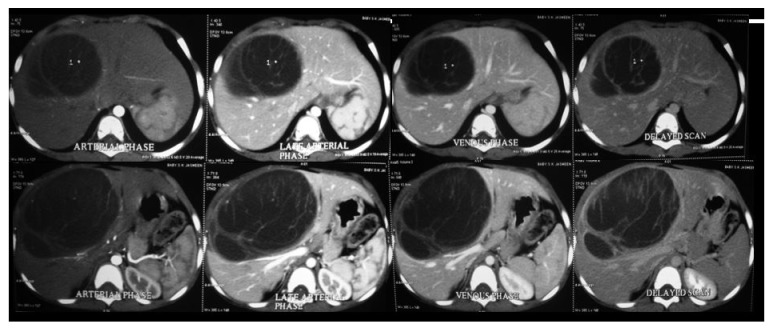
Triple phase CT scan notable for a near water attenuating cyst occupying the central sections of the liver with involvement of the middle hepatic vein. There are few clustered microcalcifications in the cyst.

**Figure 2:**
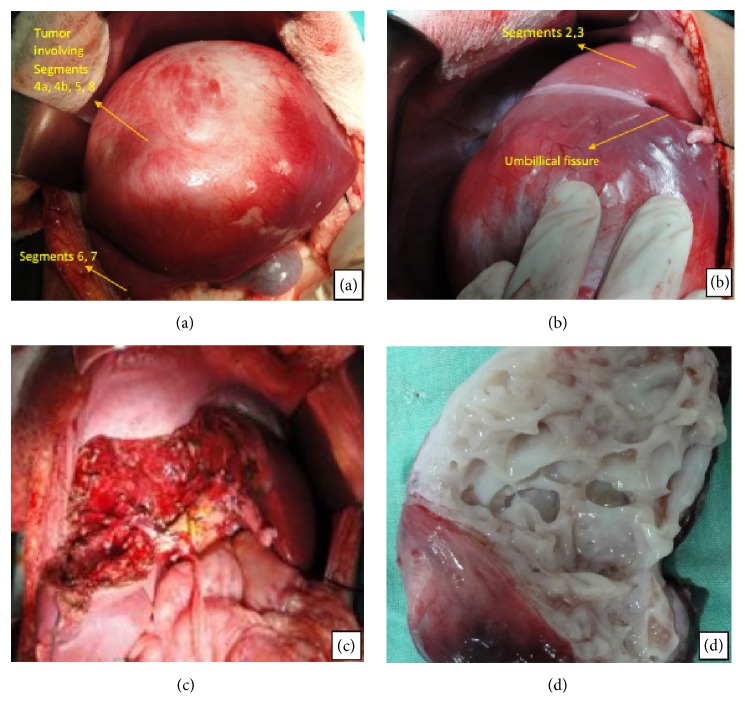
Panels (a) and (b): tumor occupying segments 4a, 4b, 5, and 8 with relative sparing of 6, 7, 2, 3, and 1. Panel (c) demonstrating resection bed of the tumor after central hepatectomy with remaining liver segments. Panel (d) demonstrates the cut section of the tumor with a Swiss cheese pattern with multiple cystic spaces traversed by septae.

A 4-year-old girl was incidentally detected to have a right upper quadrant (RUQ) mass during work-up for diarrheal illness which has since resolved. Mother denies any history of pain, jaundice, fever, or weight loss. Developmental milestones were normal. Physical exam was notable for an active child, with a firm mass from liver. Work-up revealed a normal hemogram, liver, and renal function tests. Alpha-feto protein level was 83 ng/ml. Viral markers for Hepatitis B and Hepatitis C were negative. Sonogram of the abdomen was notable for an avascular hypoechoic liver mass with septations with no mural nodules. MDCT of the liver (Figure 1) was notable for a cystic SOL with enhancing septae occupying segments 4a and 4b and abutting the middle hepatic vein. CT volumetry revealed an adequate future liver remnant for both left and right trisectionectomy. At operative exploration (Figure 2) the tumor was found to extend to segments 5 and 8. She underwent a central hepatectomy with resection of segments 4a, 4b, 5, and 8. Postoperative course was uneventful and she was discharged home on fifth postoperative day. The final histopathological diagnosis was a mesenchymal hamartoma of the liver with all margins negative for tumor involvement. One-year follow-up on the child was negative for any stigmata of recurrent disease.

## 3. Discussion

MH is considered a ductal plate malformation, wherein mesenchymal rests become isolated from the normal portal triad architecture and differentiate independently [3, 4]. The biologic behavior of these tumors varies with the relative predominance of blood vessels and bile ducts within the loose connective tissue stroma (mesenchymal) that surrounds them. Other theories gaining acceptance are regional ischemia, toxic-metabolic, and a true neoplastic etiology with most cytogenetic analysis pointing to chromosomal abnormalities involving the region 19q13.4 [1, 4].

Grossly, mesenchymal hamartoma is a well circumscribed, unencapsulated mass that can be very large with a soft, myxoid, and cystic cut surface (Figure 2). The tumor presents as a cystic structure and enlarges rapidly because of fluid accumulation. Microscopically, there is a mixture of epithelial and stromal components. The epithelial component consists mainly of tortuous and dilated bile duct elements. Cystic changes in the bile ducts could present as ectasia, pseudocysts, or lymphangiomatous malformation [5]. The stromal component is formed of spindle cells in a background that ranges from myxoid in about 50% and collagenous or hyalinized in 47% [5]. As a minor component of MH, vascular proliferation and hepatocytes were identified. Vessels consisted of small to medium sized veins or capillaries. Hepatocytes were found in cords, islands, or lobules. The hepatocytes were largely located in the periphery of the hamartoma. However, in few cases, they were widely distributed within the entire hamartomas. Transition between hepatocytes and bile ducts was also identified focally in about a third of the cases [5].

About three-quarters of MH occur in the right lobe of the liver. The rest are found in the left lobe or involve both lobes. Up to 20% of MH are pedunculated, arising from the inferior surface of the liver. Painless RUQ mass noticed by the mother or an incidental imaging finding is the usual presentation. Mechanical compression of adjacent viscera causing pain, vomiting, jaundice, or poor weight gain is described. There is no tumor marker (AFP, *β*-HCG) or liver function test that is specific for a diagnosis of MH. Normal laboratory parameters are useful in excluding other diagnoses. The usual imaging finding is a large, well-defined, heterogeneous solitary mass containing cysts of varying sizes, ranging from a few millimeters to more than 15 cm. The characteristic ultrasonography findings are multiple echogenic cysts with thin septa. On a noncontrast CT the stromal elements appear hypoattenuating and the cystic components are of near water attenuation. The mesenchymal component enhances with contrast administration [1, 2]. MR imaging appearance of mesenchymal hamartoma depends on the relative cystic versus stromal components. Solid areas may appear hypointense to adjacent liver both on T1- and T2-w images owing to fibrosis. The cystic areas are generally close to water signal intensity on T2-weighted images and demonstrate variable signal intensity on T1-weighted images, depending on the protein content of the cyst fluid. After intravenous administration of gadolinium, enhancement is mild and limited to the septa and stromal components [1, 2].

The current standard of care of these tumors is complete resection with clear margins. A formal hepatic resection or nonanatomical resection could accomplish these goals. Besides symptoms a strong indication for resection of all these tumors is a well proven association with undifferentiated embryonal sarcoma (UES), an aggressive liver tumor with a median survival of less than 1.5 years [1, 6, 7]. UES after incomplete excision of MH, coexistence of the two entities in the same tumor and similar features on gross pathology, immunohistochemistry, and cytogenetics suggest a strong association between the two and the plausible theory that MH can degenerate into UES [6, 7]. If deemed unresectable orthotropic liver transplantation or LDLT should be considered for a long term survival [8].

The practice of watchful waiting, enucleation, and marsupialization is strongly discouraged for the above-mentioned reasons [1, 6, 7, 9]. If a nonoperative management is chosen a close surveillance protocol including clinical examination and imaging (USG) is the best possible strategy to identify any malignant conversion at the earliest.

The overall mortality after liver resection for primary liver malignancies in children is 3.7% [10]. In series that specifically reported outcomes after resection for MH, the mortality rate is higher [11, 12]. Mortality is mainly from an acute event like blood loss and air embolism and from unrelated causes. Morbidity rates tend to be substantial at 30–35% [1, 10–12]. Major morbidity stems from bile leaks and bile duct strictures, postoperative infections, and incisional hernias. Most morbidity is treatable and is with no long term consequences. Operations performed at high volume centres tend to have lower mortality and morbidity rates [8].

Parental involvement in treatment decisions, heightened awareness of malignant transformation, and an expert surgical management are essential elements of high value care in treating these tumors.
